# Supplementary material for: SMAD4 Expression in Monocytes as a Potential Biomarker for Atherosclerosis Risk in Patients with Obstructive Sleep Apnea
Source: Int J Mol Sci. 2023 Apr 26;24(9):7900. doi: 10.3390/ijms24097900 (PMC10178665; doi:10.3390/ijms24097900)
Supplement: Supplementary file 1 [file ijms-24-07900-s001.zip › ijms-2310324-supplementary.pdf]

International Journal of *Molecular Sciences*

MDPI

Supplementary material

SMAD4 Expression in Monocytes as a Potential Biomarker for Atherosclerosis Risk in Patients with Obstructive Sleep Apnea

Elena Díaz-García[1,2], Aldara García-Sánchez[1,3], David Sanz-Rubio[4], Enrique Alfaro[1,2], Cristina López-Fernández[2], Raquel Casitas[1,2], Eva Mañas Baeona[3], Irene Cano-Pumarega[3], Pablo Cubero[4], Marta Marin-Oto[4], Eduardo López-Collazo[1,5], José M. Marin[4,6], Francisco García-Río[1,2,7,*], Carolina Cubillos-Zapata[1,2,*]

1 Biomedical Research Networking Centre on Respiratory Diseases (CIBERES), Madrid, Spain.
2 Respiratory Diseases Group, Respiratory Diseases Department, La Paz University Hospital, IdiPAZ, Madrid, Spain.
3 Servicio de Neumología, Hospital Universitario Ramón y Cajal, Madrid, Spain
4 Precision Medicine in Respiratory Diseases Group, Miguel Servet University Hospital – IIS Aragon, Zaragoza, Spain
5 The Innate Immune Response Group, La Paz University Hospital, IdiPAZ, Madrid, Spain
6 Department of Medicine, University of Zaragoza School of Medicine, Zaragoza, Spain
7 Faculty of Medicine, Autonomous University of Madrid, Madrid, Spain.
* Correspondence: fgr01m@gmail.com and cubilloszapata@gmail.com

Citation: Díaz-García, E.; García-Sánchez, A.; Sánz-Rubio, D.; Alfaro, E.; López-Fernández, C.; Casitas, R.; Mañas Baena, E.; Cano-Pumarega, I.; Cubero, P.; Marin-Oto, M.; et al. SMAD4 Expression in Monocytes as a Potential Biomarker for Atherosclerosis Risk in Patients with Obstructive Sleep Apnea. *Int. J. Mol. Sci.* **2023**, *24*, 7900. https://doi.org/10.3390/ijms24097900

Academic Editor: Michele Samaja

Received: 13 March 2023
Revised: 14 April 2023
Accepted: 25 April 2023
Published: 26 April 2023

Copyright: © 2023 by the authors. Licensee MDPI, Basel, Switzerland. This article is an open access article distributed under the terms and conditions of the Creative Commons Attribution (CC BY) license (https://creativecommons.org/licenses/by/4.0/).

1. Supplementary Methods.

1.1. Study subjects

This study includes subjects belonging to the Epigenetic Status and Subclinical Atherosclerosis in Obstructive Sleep Apnea (EPIOSA, NCT02131610) cohort [1], a 5-yr noninterventional longitudinal prospective study conducted at the Sleep Clinic of the Hospital Universitario Miguel Servet (Zaragoza, Spain) to identify biomarkers associated with prevalent and progression of subclinical atherosclerosis in individuals with OSA. Inclusion criteria were the following: adults aged 20-60 years; AHI ≥ 10 events per hour of sleep (OSA group) and AHI < 5 (control group); willingness to participate in the study and complying with the study by signing a written informed consent; and available for study visits over 5 years. The exclusion criteria were: alcohol abuse, arterial blood hypertension (arterial blood pressure: ≥140 mmHg systolic and/or ≥90 mmHg diastolic or taking antihypertensive medication), low high-density lipoprotein (HDL) cholesterol (126 mg/dl or taking anti diabetic medications, other metabolic diseases, present or past history of vascular diseases, including myocardial infarction, angina, coronary artery procedures (coronary artery bypass graft or percutaneous coronary intervention), aneurysm, transient ischemic attack or stroke, autoimmune diseases, past or present history of malignancies, chronic inflammatory diseases, chronic infectious diseases, chronic respiratory diseases, morbid obesity (body mass index ≥40 kg/m2), any chronic oral therapy, sleep disorders other than OSA, having undergone upper airway surgery and having previously use CPAP therapy. All participants provided written informed consent and the study has been approved by the Regional Institutional Review Board of Aragon (IACS), Spain (project 03/2013). Anthropometric and clinical data were obtained at recruitment using specific questionnaires and standard measurements [1]. A home sleep test, which included continuous recording of airflow from a nasal pressure cannula, thoracic-abdominal motion, oxygen saturation, snoring and body position, was performed as previously reported [1]. Records were analyzed using American Academy of Sleep Medicine guidelines [2] by trained personnel and subjects were allocated according to their apnea-hypopnea index (AH) as OSA (AHI ≥ 10 events/hour of recording time) or control (AHI < 5). Common

carotid intima-media thickness (IMT) was assessed using the Philips IU22 ultrasound system (Philips Healthcare, Bothell, WA, USA). Ultrasound images were acquired with linear highfrequency 2-dimensional probes (Philips Transducer L9-3, Philips Healthcare), using the Bioimage Study protocol for the carotid arteries [3]. Examination of the carotid territory included the terminal portion (10 mm) of the common carotid, the bulb, and the initial portion (10 mm) of the internal and external carotid arteries and three measurements were performed in end diastole. Subclinical atherosclerosis was defined in subjects with a IMT greater than the upper limit (75 percentile) of the normal distribution of the maximum IMT by segments of age groups as reported in our local healthy population [4]. Subjects with at least a carotid plaque, defined as a local structure that protrudes into the lumen of the carotid artery at least 0.5 mm or ≥50% thicker than the surrounding, were excluded of this analysis. Finally, the study groups were constituted by OSA patients (OSA) and healthy subjects (HS), with or without evidence of early subclinical atherosclerosis (non-eSA and eSA, respectively). Whole blood samples are drawn using a 21G butterfly needle into EDTA tubes. Plasma samples and buffy coats were obtained by gradient centrifugation (3000 rpm, 5min) and stored at -80ºC for further analysis. The present study was only performed with baseline samples.

In the RC cohort, recently diagnosed severe OSA patients were consecutively recruited from the Pneumology Service of Ramón y Cajal University Hospital, Madrid, Spain. Patients aged between 40 and 65 years with an apnea-hypopnea index (AHI) > 30 events/h were included in the study. Exclusion criteria were the following: previous or current treatment with oxygen or mechanical ventilation; underweight patients (body mass index [BMI] <18.5 Kg/m2) or those with morbid obesity (BMI >40 Kg/m2); history of respiratory disease, including chronic obstructive pulmonary disease, asthma or respiratory failure; any infectious disease in the previous 3 months; and the use of inhaled or systemic corticosteroids or other anti-inflammatory drugs. The subjects were classified as current smokers (defined as daily smoking of any number of cigarettes), former smokers (defined as patients who stopped smoking at least 6 months before study inclusion), and nonsmokers. As a control group, healthy volunteers were selected who were homogeneous in sex, age, smoking habit and BMI. None of these volunteers were being treated with any type of medication, and the diagnosis of OSA was ruled out by respiratory polygraphy. The study was approved by the Hospital Universitario La Paz Ethics Committee (PI-3643) and extended to Ramón y Cajal University Hospital.

2. Supplementary Tables

Table S1. SMAD4 mRNA levels correlation with hypoxemia parameters.

	**SMAD4 mRNA (EPIOSA)**			**SMAD4 mRNA (RC)**		
	**r**	**p-value**	**n**	**r**	**p-value**	**n**
**AHI, events/h**	0.457	0.005	36	0.28	0.02	50
**ODI, events/h**	0.557	0.004	25	0.23	0.07	50
**CT90, %**	0.422	0.012	36	-	-	-
**Mean nocturnal SaO_2, %**	-0.355	0.033	35	-0.27	0.04	50
**Low nocturnal SaO_2, %**	-0.360	0.033	35	-0.28	0.03	50

* Correlations were performed by Pearson's correlation test. Abbreviations: AHI=apnea-hypopnea index; ODI=oxygen desaturation index, CT90= Time recorded with SaO2<90%, SaO2=oxyhemoglobin.

3. Supplementary Figures

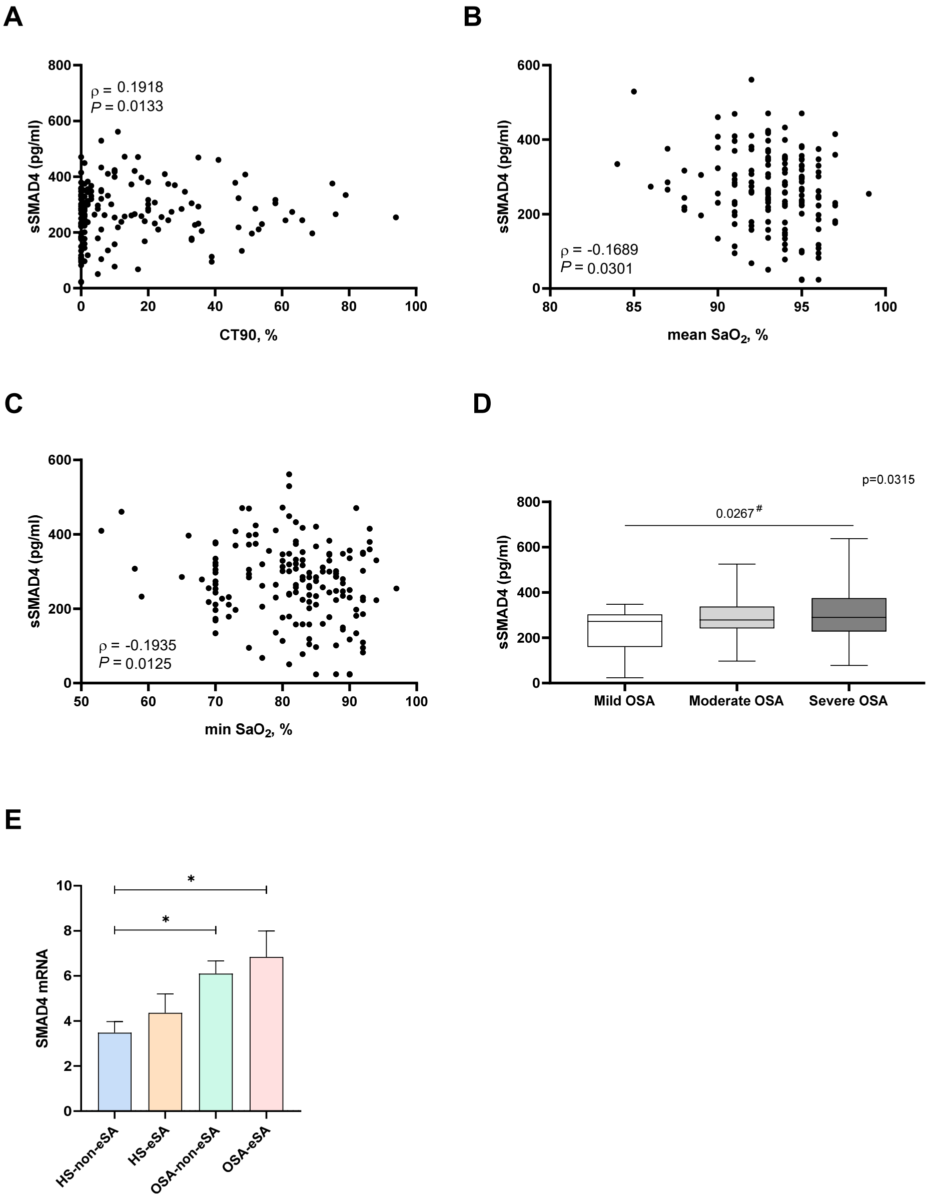

Figure S1. (A-C) Correlation between sSMAD4 plasma concentration and (A) Time recorded with SaO2<90% [CT90] (n=165), (B) mean nocturnal oxyhemoglobin saturation [mean SaO2] (n=165) and (C) lowest nocturnal oxyhemoglobin saturation [min SaO2] (n=165). Spearman's correlation coefficients (ρ) and p-values are shown. (D) Box-and-whisker plots depicting the distribution of plasma levels of sSMAD4 in EPIOSA cohort subjects according to their apnea-hypopnea index (AHI) (mild, AHI >5, <15; moderate, AHI. ≥15, <30, severe AHI. ≥30. Data are presented as median (interquartile range), maximum and minimum values, and overall comparisons were performed using the Kruskal-Wallis test. #: P-values corresponding to the post hoc comparisons between groups. OSA: obstructive sleep apnea, (E) SMAD4 mRNA levels in PBMCs from EPIOSA subjects: control subjects without early subclinical atherosclerosis (HS-non-eSA, n=9) or with early subclinical atherosclerosis (HS-eSA, n=8) and in OSA patients without (OSA-non-eSA, n=10) or with early subclinical atherosclerosis (OSA-eSA, n=9) determined by qPCR. Data are presented as mean ± standard error of the mean (SEM). Comparisons between groups were performed by Kruskal-Wallis test. *: P<0.05.

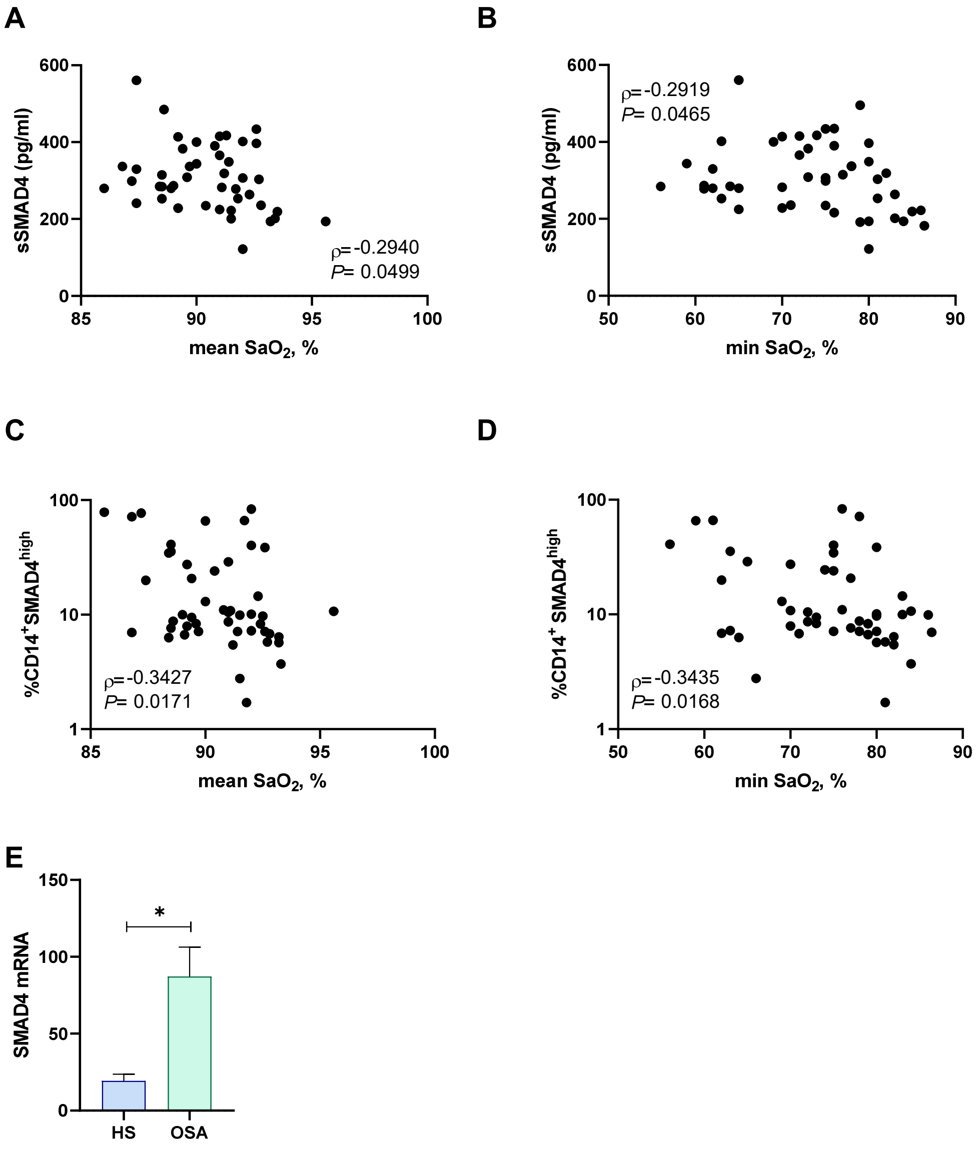

Figure S2. (A-B) Correlation between sSMAD4 plasma concentration from RC subjects and (A) mean nocturnal oxyhemoglobin saturation [mean SaO2] (n=50) and (B) lowest nocturnal oxyhemoglobin saturation [min SaO2] (n=50). (C-D) Correlation between monocyte intracellular SMAD4 expression from RC subjects and (C) mean nocturnal oxyhemoglobin saturation [mean SaO2] (n=50) and (D) lowest nocturnal oxyhemoglobin saturation [min SaO2] (n=50). Spearman's correlation coefficients (ρ) and p-values are shown. (E) SMAD4 mRNA levels in monocytes from RC subjects: control subjects (HS, n=20) and OSA patients (OSA, n=50) determined by qPCR. Data are presented as mean ± standard error of the mean (SEM). Comparisons between groups were performed by unpaired T-test. *: P<0.05.

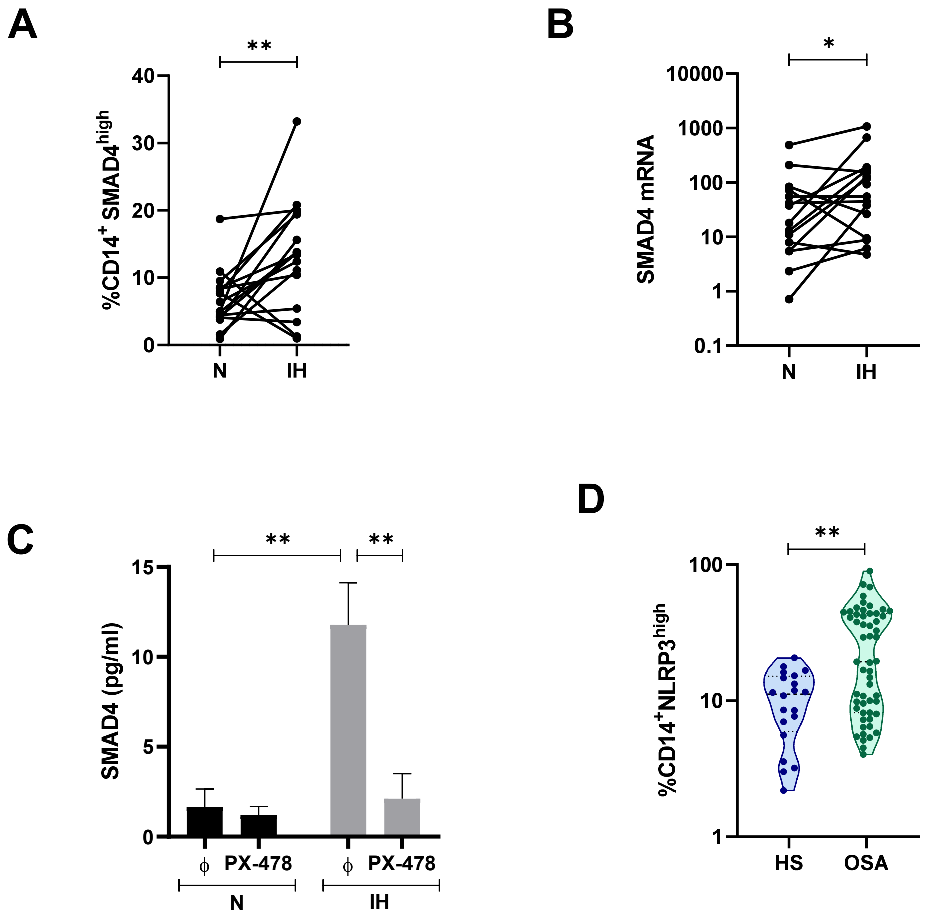

Figure S3. (A) SMAD4 intracellular expression determined by flow cytometry and (B) SMAD4 mRNA levels determined by qPCR in monocytes from healthy volunteers (n=16) subjected to normoxia conditions (N) or intermittent hypoxia conditions (IH) for 16 hours. Comparisons between groups were performed by Wilcoxon test. (C) SMAD4 concentration determined by ELISA in the supernatant from healthy volunteers' monocytes (n=7) treated or not with a specific inhibitor for HIF-1α (PX-478 30μM) and/or subjected to intermittent hypoxia conditions for 16 hours. Comparisons between groups were performed by Two-way ANOVA with Bonferroni's correction for multiple comparison tests. (D) NLRP3 intracellular expression in monocytes from RC cohort subjects: control subjects (HS, n=20) or OSA patients (OSA, n=50) determined by flow cytometry. Comparison between groups was performed by Mann-Whitney U-test. *: P<0.05, **: P<0.01.

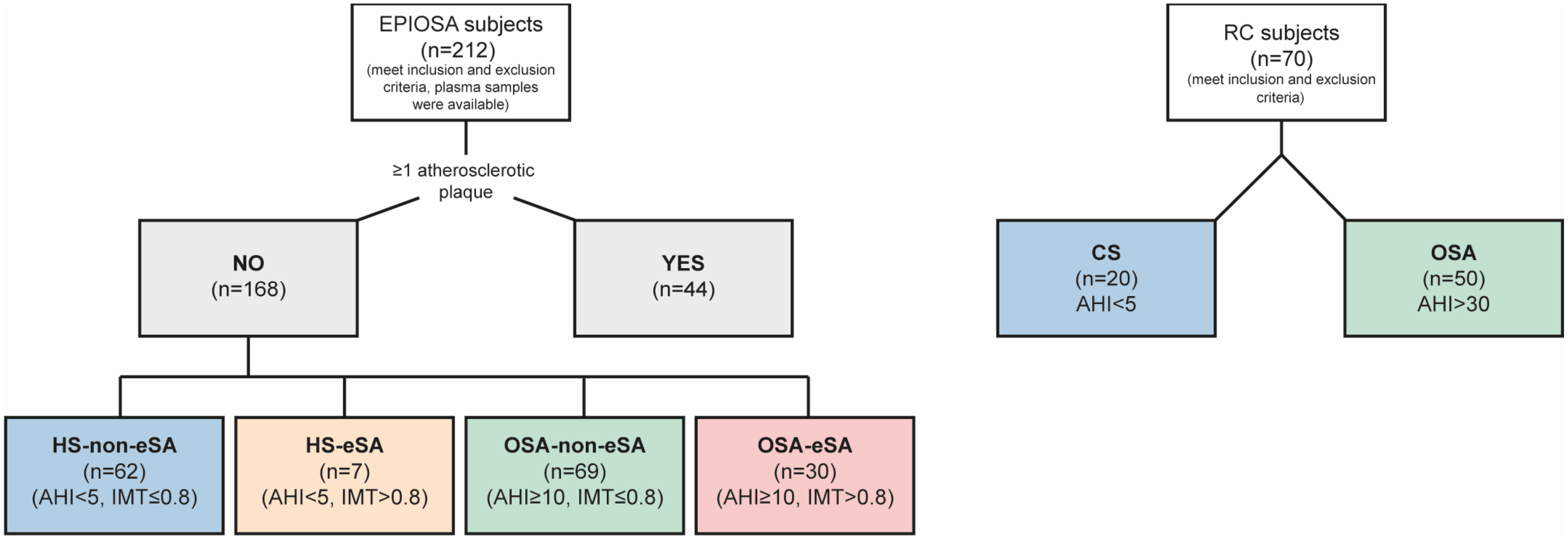

Figure S4. Flow chart of included participants. Two independent cohorts were included. In EPIOSA cohort, subjects were classified according to the presence of atherosclerotic plaques (NO, n=168; YES, n=44). Subjects without any atherosclerotic plaque were then classified according to apnea-hypopnea index (AHI) and intima-media thickness (IMT). Thus, four groups were established: control subjects without early subclinical atherosclerosis (HS-non-eSA, AHI<5, IMT≤0.8); controls subjects with early subclinical atherosclerosis (HS-non-eSA, AHI<5, IMT>0.8); obstructive sleep apnea subjects without early subclinical atherosclerosis (OSA-non-eSA, AHI≥10, IMT≤0.8); obstructive sleep apnea subjects with early subclinical atherosclerosis (OSA-eSA, AHI≥10, IMT>0.8). In the RC cohort subjects were classified according to their AHI in control subjects (AHI<5) or OSA subjects (OSA AHI>30).

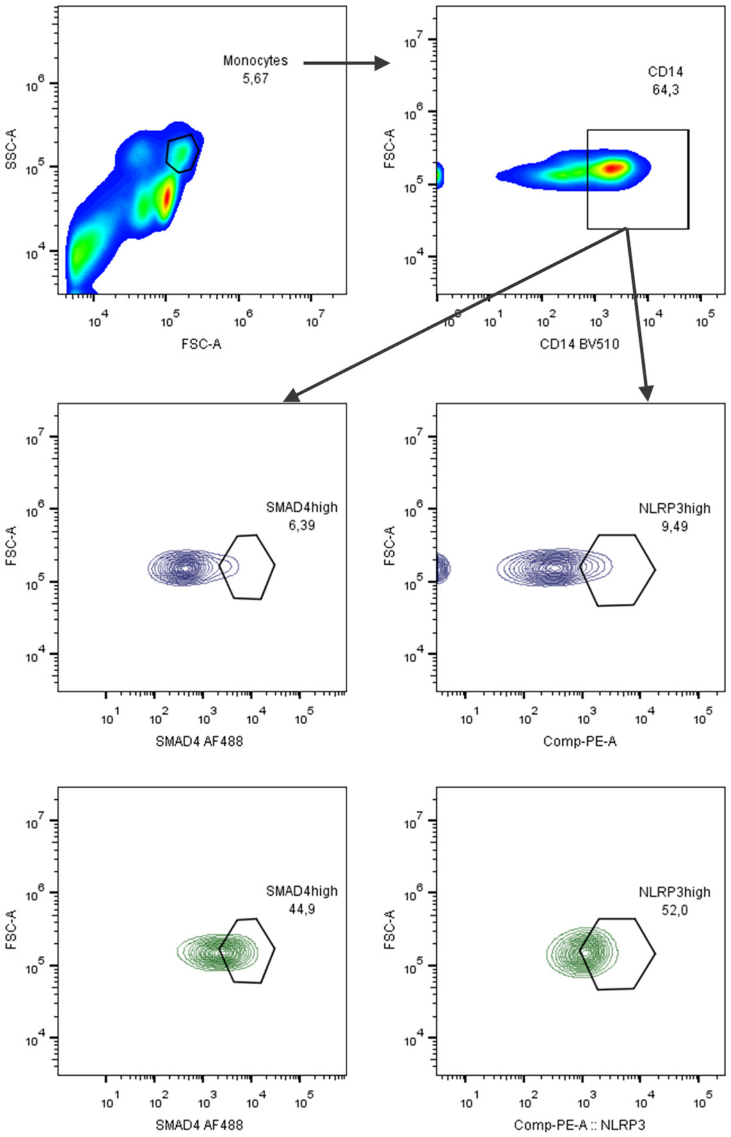

Figure S5. Gating strategy for cytometry analysis in the RC cohort. Blue plots corresponding to HS subjects. Green plots corresponding to OSA subjects.

REFERENCES

1. Marin, J.M.; Artal, J.; Martin, T.; Carrizo, S.J.; Andrés, M.; Burriel, I.M.; Bolea, R.; Sanz, A.; Varona, L.; Godino, J.; et al. Epigenetics modifications and Subclinical Atherosclerosis in Obstructive Sleep Apnea: The EPIOSA study. BMC Pulm. Med. **2014**, 14, 114–114, https://doi.org/10.1186/1471-2466-14-114

2. Berry, R.B.; Budhiraja, R.; Gottlieb, D.J.; Gozal, D.; Iber, C.; Kapur, V.K.; Marcus, C.L.; Mehra, R.; Parthasarathy, S.; Quan, S.F.; et al. Rules for Scoring Respiratory Events in Sleep: Update of the 2007 AASM Manual for the Scoring of Sleep and Associated Events. Deliberations of the Sleep Apnea Definitions Task Force of the American Academy of Sleep Medicine. *J. Clin. Sleep Med.* **2012**, *8*, 597–619, https://doi.org/10.5664/jcsm.2172.

3. Muntendam, P.; McCall, C.; Sanz, J.; Falk, E.; Fuster, V. The BioImage Study: Novel approaches to risk assessment in the primary prevention of atherosclerotic cardiovascular disease—study design and objectives. *Am. Hear. J.* **2010**, *160*, 49–57.e1, doi:10.1016/j.ahj.2010.02.021.

4. Jarauta, E.; Mateo-Gallego, R.; Bea, A.; Burillo, E.; Calmarza, P.; Civeira, F. Carotid Intima-Media Thickness in Subjects With No Cardiovascular Risk Factors. **2010**, *63*, 97–102, https://doi.org/10.1016/s1885-5857(10)70014-1
